# Burden and correlates of cognitive impairment among hypertensive patients in Tanzania: a cross-sectional study

**DOI:** 10.1186/s12883-021-02467-3

**Published:** 2021-11-08

**Authors:** Pedro Pallangyo, Zabella S. Mkojera, Makrina Komba, Lucy R. Mgopa, Smita Bhalia, Henry Mayala, Salma Wibonela, Nsajigwa Misidai, Happiness J. Swai, Jalack Millinga, Ester Chavala, Peter R. Kisenge, Mohamed Janabi

**Affiliations:** 1 PédPäl Research Initiative, P.O Box 65066, Dar es Salaam, Tanzania; 2 Directorate of Cardiology, Jakaya Kikwete Cardiac Institute, P.O Box 65141, Dar es Salaam, Tanzania; 3 Department of Psychiatry and Mental Health, Muhimbili University of Health and Allied Sciences, P.O Box 65001, Dar es Salaam, Tanzania; 4 Directorate of Clinical Support Services, Jakaya Kikwete Cardiac Institute, P.O Box 65141, Dar es Salaam, Tanzania; 5 Directorate of Nursing, Jakaya Kikwete Cardiac Institute, P.O Box 65141, Dar es Salaam, Tanzania

**Keywords:** Cognitive dysfunction, Cognitive impairment, Cognitive decline, Cognitive deficits, Arterial hypertension, Systemic hypertension, Hypertension, Elevated blood pressure, High blood pressure, Sub Saharan Africa, Jakaya Kikwete cardiac institute, Tanzania

## Abstract

**Background:**

The evolution of cognitive impairment of vascular origin is increasingly becoming a prominent health threat particularly in this era where hypertension is the leading contributor of global disease burden and overall health loss. Hypertension is associated with the alteration of the cerebral microcirculation coupled by unfavorable vascular remodeling with consequential slowing of mental processing speed, reduced abstract reasoning, loss of linguistic abilities, and attention and memory deficits. Owing to the rapidly rising burden of hypertension in Tanzania, we sought to assess the prevalence and correlates of cognitive impairment among hypertensive patients attending a tertiary cardiovascular hospital in Tanzania.

**Methodology:**

A hospital-based cross-sectional study was conducted at Jakaya Kikwete Cardiac Institute, a tertiary care public teaching hospital in Dar es Salaam, Tanzania between March 2020 and February 2021. A consecutive sampling method was utilized to recruit consented hypertensive outpatients during their scheduled clinic visit. General Practitioner Assessment of Cognition (GPCOG) Score was utilized in the assessment of cognitive functions. All statistical analyses utilized STATA v11.0 software. Pearson Chi square and Student’s T-test were used to compare categorical and continuous variables respectively. Logistic regression analyses were used to assess for factors associated with cognitive impairment. Odd ratios with 95% confidence intervals and *p*-values are reported. All tests were 2-sided and *p* < 0.05 was used to denote a statistical significance.

**Results:**

A total of 1201 hypertensive patients were enrolled in this study. The mean age was 58.1 years and females constituted nearly two-thirds of the study population. About three quarters had excess body weight, 16.6% had diabetes, 7.7% had history of stroke, 5.7% had heart failure, 16.7% had renal dysfunction, 53.7% had anemia, 27.7% had hypertriglyceridemia, 38.5% had elevated LDL, and 2.4% were HIV-infected. Nearly two-thirds of participants had uncontrolled blood pressure and 8.7% had orthostatic hypotension. Overall, 524 (43.6%) of participants had cognitive impairment. During bivariate analysis in a logistic regression model of 16 characteristics, 14 parameters showed association with cognitive functions. However, after controlling for confounders, multivariate analysis revealed ≤primary education (OR 3.5, 95%CI 2.4–5.2, *p* < 0.001), unemployed state (OR 1.7, 95%CI 1.2–2.6, *p* < 0.01), rural habitation (OR 1.8, 95%CI 1.1–2.9, *p* = 0.01) and renal dysfunction (OR 1.7, 95%CI 1.0–2.7, *p* = 0.04) to have independent association with cognitive impairment.

**Conclusion:**

This present study underscore that cognitive decline is considerably prevalent among individuals with systemic hypertension. In view of this, it is pivotal to incorporate cognitive assessment in routine evaluation of hypertensive patients.

**Supplementary Information:**

The online version contains supplementary material available at 10.1186/s12883-021-02467-3.

## Background

Systemic arterial hypertension, the leading cause of global disease burden and overall health loss, affects over two-fifths of the adult population worldwide[1–4]. Given the progressive ageing of the world population and considering the rapidly growing prevalence of uncontrolled hypertension, the evolution of cognitive impairment is increasingly becoming a prominent health threat. The World Health Organization (WHO) estimates that about two-thirds of the cerebrovascular disease is attributable to elevated blood pressure[5]. Furthermore, accruing epidemiological and mechanistic evidence suggests that hypertension is a risk factor for the onset and progression of cognitive impairment, vascular dementia and Alzheimer’s disease[6–63]. In view of its pivotal role in cognitive impairment particularly of vascular origin, the WHO has set a global target to relatively reduce 25% of the hypertension burden by 2025 as a fundamental measure to reduce the risk of cognitive decline[64].

Although our current understanding predominantly emphasize on the well-established effect of high blood pressure in the development of stroke, the impact on cognitive consequences appears to be independent of stroke[38, 46]. The deleterious effects of hypertension on the brain targets the cerebral blood vessels with resultant structural and functional cerebrovascular alterations including white matter damage, frontal lobe dysfunction, small vessel disease, lacunar strokes, cerebral microhemorrhages, arteriosclerosis, silent brain infarcts, and brain atrophy[65–76]. As a consequence, hypertension is associated with reduced abstract reasoning (executive dysfunction), slowing of mental processing speed, loss of linguistic abilities, and attention and memory deficits[10, 77–80]. Owing to the paucity of data regarding the association between arterial hypertension and cognitive decline particularly in resource-limited settings, this present study aimed to explore the burden and correlates of cognitive impairment among hypertensive patients attending a tertiary cardiovascular hospital in Tanzania.

## Methods

### Study design, recruitment process, and definition of terms

This hospital-based cross-sectional study was conducted at Jakaya Kikwete Cardiac Institute (JKCI), a tertiary care public teaching hospital in Dar es Salaam, Tanzania between March 2020 and February 2021. A consecutive sampling method was utilized to recruit consented hypertensive outpatients during their scheduled clinic visit. A structured questionnaire bearing questions pertaining to sociodemographic and clinical characteristics, measurement of key vitals (blood pressure [BP], blood sugar, height, and weight) was used during participants’ interviews. Social activity, sleeping habits, vision/hearing status, mental health history, seizure disorders, dietary intake and history of stroke were self-reported.

The General Practitioner Assessment of Cognition (GPCOG), a cognitive impairment screening tool copyrighted by the University of New South Wales was utilized in the assessment of cognitive impairment. The GPCOG has been validated for use in a wide variety of populations including hypertension and resistant hypertension subpopulations. With a sensitivity and specificity for the English GPCOG ranging from 0.81 to 0.98 and 0.72 to 0.95, respectively; the GPCOG performed at least as well as, if not better, than the widely-used cognitive screens such as the Mini-Mental State Examination (MMSE) or the Abbreviated Mental Test (AMT)[81]. The GPCOG consists of two parts: (i) a 9-items cognitive assessment (i.e. time orientation, visuospatial functioning, information and recall) conducted with the patient whereby a score of 0–4 indicates cognitive impairment, a score of 5–8 signify inconclusive results and a score of 9 implies no significant cognitive impairment and warrants no further testing and (ii) a 6-questions informant questionnaire, which is only performed if the results of the cognitive assessment are inconclusive. If the patient scores 0–3 out of 6, cognitive impairment is indicated[81].

Physical activity was assessed using the Physical Activity Vital Sign (PAVS)[82] questionnaire whereby reported moderate-vigorous physical activity of 0 min/week, < 150 min/week, or ≥ 150 min/week was used to categorize participants as inactive, underactive or active respectively. We defined underweight as BMI < 18.5 kg/m^2^, normal: BMI 18.5–24.9 kg/m^2^, overweight: BMI 25.0–29.9 kg/m^2^ and obese: BMI ≥30.0 kg/m^2^[83]. Individuals who smoked at least 1 cigarette in the past 6 months were regarded as current smokers, those who last smoked over 6 months or self-reported quitting smoking were considered ex-smokers and those who never smoked were regarded as never-smokers. Alcohol drinking was defined as at least a once consumption every week. Social activeness was assessed through participants’ self-assessment (i.e. active vs inactive) regarding their participation in important social activities (i.e. weddings, burial ceremonies, and traditional festivals).

Hypertension was defined as SBP ≥140 mmHg or DBP ≥90 mmHg, and/or use of BP lowering agents[84]. Moreover, SBP ≥140 mmHg or DBP ≥90 mmHg was used to define uncontrolled BP. Orthostatic hypotension was defined by a decrease in SBP ≥20 mmHg or DBP ≥10 mmHg within 3 min of standing when compared to the sitting measurements. Diabetes was diagnosed using a glycated hemoglobin (HbA1c) of ≥6.5% and/or fasting blood glucose (FBG) ≥7 mmol/L or use of glucose-lowering agents[85]. A 2-dimensional echocardiography was utilized for the diagnosis heart failure. Renal functions were estimated using the Modification of Diet in Renal Disease (MDRD) equation and renal dysfunction was defined by an estimated glomerular filtration rate (eGFR) value of < 60 mL/min/1.73 m^2^[86].WHO criteria for anemia i.e. hemoglobin (Hb) concentration of < 13.0 g/dL for males and < 12.0 g/dL for females was used to diagnose anemia[87]. Triglycerides and low-density lipoprotein (LDL) cut-off levels of 1.7 mmol/L[88] and 3.5 mmol/L[89] respectively were used to categorize hypertriglyceridemia and elevated LDL respectively. Uric acid levels of 480 μmol/L and 360 μmol/L were used to denote hyperuricemia among males and females respectively[90].

### Statistical analysis

All statistical analyses utilized STATA v11.0 software. Summaries of continuous variables and categorical variables are presented as are presented as means (± SD) and frequencies (percentages) respectively. Pearson Chi square and Student’s T-test were used in comparison of categorical and continuous variables respectively. Logistic regression analyses were used to assess for factors associated with cognitive impairment. Stepwise and forward selection procedure was used to add and assess the statistically significant variables in the multivariate regression model. The multivariate model was fitted with baseline covariates associated with cognitive impairment by bivariate analysis at the < 0.05 significance level. Odd ratios (OR) with 95% confidence intervals and *p*-values are reported. All tests were 2-sided and *p* < 0.05 was used to denote a statistical significance.

## Results

### Study population characteristics

A total of 1201 hypertensive patients were enrolled in this study. Table 1, displays sociodemographic and clinical characteristics of study participants. The mean age was 58.1 years and just over a half of all participants were aged 60 years or more. Nearly two-thirds (64.4%) of all participants were female, 71.2% were married, 66.3% had a regular income generating activity, and 65.0% had attained at most primary education. Large majority (83.1%) resided in urban areas and 92.0% lived with their families. About 1% were current smokers, 6.6% consumed alcohol, and 86.3% were socially active. Over a quarter (27.3%) of all participants were physically active and about three quarters (75.7%) were overweight or obese. Over one-third had insomnia (36.3%), 16.6% had diabetes, 7.7% had history of stroke, 5.7% had heart failure, 16.7% had renal dysfunction, 53.7% had anemia, 32.4% had hyperuricemia, 27.7% had hypertriglyceridemia, 38.5% had elevated LDL, and 2.4% were HIV-infected. Nearly two-thirds (65.4%) of participants had uncontrolled BP and 8.7% had orthostatic hypotension.

**Table 1:** Sociodemographic and clinical characteristics of study participants by cognitive status

Characteristic	All ***N*** = 1201	Preserved Cognition ***n*** = 677	Cognitive Dysfunction ***n*** = 524	***p*** - value
**Age**				
Mean (SD)	58.1 (11.3)	55.7 (10.9)	61.1 (11.1)	**< 0.001**
< 60 years	597 (49.7%)	392 (57.9%)	205 (39.1%)	
≥60 years	604 (50.3%)	285 (42.1%)	319 (60.9%)	**< 0.001**
**Sex**				
Male	427 (35.6%)	269 (39.7%)	158 (30.1%)	
Female	774 (64.4%)	408 (60.3%)	366 (69.9%)	**< 0.001**
**Education level**				
No formal	74 (06.2%)	16 (02.3%)	58 (11.1%)	**< 0.001**
Primary	706 (58.8%)	345 (51.0%)	361 (68.9%)	**< 0.001**
Secondary	279 (23.2%)	203 (30.0%)	76 (14.5%)	**< 0.001**
University	142 (11.8%)	113 (16.7%)	29 (05.5%)	**< 0.001**
**Marital status**				
Single	40 (03.3%)	23 (03.4%)	17 (03.2%)	0.85
Married	855 (71.2%)	517 (76.4%)	338 (64.5%)	**< 0.001**
Divorced	68 (05.7%)	36 (05.3%)	32 (06.1%)	0.55
Widowed	238 (19.8%)	101 (14.9%)	137 (26.2%)	**< 0.001**
**Occupation**				
Student	3 (0.3%)	2 (0.3%)	1 (0.2%)	0.91
Unemployed	292 (24.3%)	97 (14.3%)	195 (37.2%)	**< 0.001**
Self employed	504 (42.0%)	313 (46.2%)	191 (36.5%)	**< 0.001**
Employed	194 (16.1%)	141 (20.9%)	53 (10.1%)	**< 0.001**
Retired	208 (17.3%)	124 (18.3%)	84 (16.0%)	0.30
**Residence**				
Urban	998 (83.1%)	593 (87.6%)	405 (77.3%)	
Rural	203 (16.9%)	84 (12.4%)	119 (22.7%)	**< 0.001**
**Cigarette smoking**				
Never	1042	593 (87.6%)	449 (85.7%)	0.34
Ex-smoker	(86.8%)	74 (10.9%)	72 (13.7%)	0.14
Current	146 (12.2%) 13 (01.0%)	10 (01.5%)	3 (0.6%)	0.14
**Alcohol drinking**				
Never	705 (58.7%)	374 (55.2%)	331 (63.2%)	**< 0.01**
Past	417 (34.7%)	243 (35.9%)	174 (33.2%)	0.33
Current	79 (06.6%)	60 (08.9%)	19 (03.6%)	**< 0.001**
**Physical activity**				
Mean (min/week)	97.5 (102.5)	104.2 (104.8)	88.7 (98.9)	**< 0.01**
Inactive	240 (20.0%)	115 (17.0%)	125 (23.9%)	**< 0.01**
Underactive	635 (52.9%)	366 (54.0%)	269 (51.3%)	0.35
Active	326 (27.1%)	196 (29.0%)	130 (24.8%)	0.10
**Socially active**	1037 (86.3%)	612 (90.4%)	425 (81.1%)	**< 0.001**
**Comorbidities:**				
Stroke	92 (07.7%)	43 (06.4%)	49 (09.4%)	**0.05**
Significant head injury	60 (05.0%)	33 (04.9%)	27 (05.2%)	0.81
Seizure disorder	3 (0.3%)	2 (0.3%)	1 (0.2%)	0.73
Psychiatric illness	22 (01.8%)	14 (02.1%)	8 (01.5%)	0.44
Vision/Hearing impairment	583 (48.5%)	312 (46.1%)	271 (51.7%)	**0.05**
Insomnia	436 (36.3%)	214 (31.6%)	222 (42.4%)	**< 0.001**
Anemia^a^	281 (53.7%)	145 (50.7%)	136 (57.4%)	0.13
Iron deficiency anemia^a^	76 (14.5%)	38 (13.3%)	38 (16.0%)	0.38
Anemia of chronic illness^a^	114 (21.8%)	61 (21.3%)	53 (22.4%)	0.76
Dyslipidemia				
Elevated Triglycerides^a^	94 (27.7%)	48 (24.7%)	46 (31.7%)	0.16
Elevated LDL^a^	139 (38.5%)	86 (42.0%)	53 (34.0%)	0.12
Low HDL^a^	144 (49.0%)	72 (44.2%)	72 (55.0%)	0.07
Hyperuricemia^a^	97 (32.4%)	56 (32.9%)	41 (31.8%)	0.83
Diabetes mellitus	199 (16.6%)	105 (15.5%)	94 (17.9%)	0.26
HIV	29 (02.4%)	14 (02.1%)	15 (02.9%)	0.38
Renal dysfunction^a^	103 (16.7%)	46 (13.5%)	57 (20.6%)	**< 0.01**
Heart Failure	68 (05.7%)	36 (05.3%)	32 (06.1%)	0.56
**Body Mass Index**				
Mean (SD)	29.5 (6.1)	29.9 (6.0)	28.9 (6.2%)	**< 0.01**
Underweight	15 (01.3%)	6 (0.9%)	9 (1.7%)	0.20
Normal	277 (23.1%)	138 (20.4%)	139 (26.5%)	**0.01**
Overweight	392 (32.6%)	222 (32.8%)	170 (32.4%)	0.88
Obese	517 (43.1%)	311 (45.9%)	206 (39.3%)	**0.02**
**Blood Pressure**				
Controlled BP	416 (34.6%)	233 (34.4%)	183 (34.9%)	0.86
Postural hypotension	104 (08.7%)	47 (06.9%)	57 (10.9%)	**< 0.001**

^a^: represents a subset of participants with respective lab results

### Prevalence of cognitive impairment

Overall, 524 (43.6%) of participants had cognitive impairment. Compared to participants with preserved cognition, individuals with cognitive impairment were older (i.e. mean age 61.1 vs 55.7 years, *p* < 0.001) and had a higher proportion of those aged ≥60 years (i.e. 60.9% vs 42.1%, *p* < 0.001). Females comprised a higher proportion of the cognitive impairment group, 69.9% vs 60.3%, *p* < 0.001. There was a higher proportion of participants with ≤ primary education level (80.0% vs 53.3%, p < 0.001), unmarried status (35.5% vs 23.6%, p < 0.001), no regular income generating activity (53.4% vs 32.9%, p < 0.001) and rural residents (22.7% vs 12.4%, p < 0.001) in the group with impaired cognitive functions. Moreover, physically and socially inactive participants were significantly higher in the group with cognitive impairment, i.e. 23.9% vs 17.0%, *p* < 0.01 and 18.9% vs 9.6%, p < 0.001 respectively. Furthermore, participants with cognitive impairment displayed a higher proportion of insomnia (42.4% vs 31.6%, *p* < 0.001), orthostatic hypotension (10.9% vs 6.9%, p < 0.001) and renal dysfunction (20.6% vs 13.5%, *p* < 0.01).

### Correlates of cognitive impairment

Table 2 displays the results of logistic regression analyses for factors associated with cognitive impairment. During bivariate analysis in a logistic regression model of 16 characteristics, 14 characteristics i.e. age ≥ 60 years, female sex, ≤primary education, single/divorced/widowed status, unemployed/retired state, rural residence, non-drinker, social inactivity, vision/hearing deficits, history of stroke, insomnia, renal dysfunction, excess body weight and postural hypotension showed association with cognitive functions. However, after controlling for confounders, a multivariate analysis revealed ≤primary education (OR 3.5, 95%CI 2.4–5.2, p < 0.001), unemployed/retired state (OR 1.7, 95%CI 1.2–2.6, p < 0.01, rural residence (OR 1.8, 95%CI 1.1–2.9, *p* = 0.01) and renal dysfunction (OR 1.7, 95%CI 1.0–2.7, *p* = 0.04) to have independent association with cognitive impairment.

**Table 2:** Logistic regression analyses for factors associated with cognitive impairment

Characteristic	Comparison	OR	95% CI	p - value	Adj.OR	95% CI	p - value
Age ≥ 60 years	< 60 years	2.1	1.7–2.7	**< 0.001**	1.3	0.8–1.9	0.26
Female	Male	1.5	1.2–1.9	**0.001**	1.2	0.8–1.8	0.51
≤Primary Education	≥ Secondary Education	3.5	2.7–4.5	**< 0.001**	3.5	2.4–5.2	**< 0.001**
Single/Divorced/Widowed	Married	1.8	1.4–2.3	**< 0.001**	1.1	0.8–1.7	0.50
Unemployed/Retired	Self-employed/Employed	2.3	1.8–3.0	**< 0.001**	1.7	1.2–2.6	**< 0.01**
Rural	Urban	2.1	1.5–2.8	**< 0.001**	1.8	1.1–2.9	**0.01**
Current alcohol drinker	Non drinker	0.7	0.6–0.9	**< 0.01**	0.8	0.5–1.1	0.16
Inactive/underactive	Physically active	1.2	1.0–1.6	0.11	–	–	–
Socially inactive	Socially active	2.2	1.6–3.1	**< 0.001**	1.1	0.7–1.8	0.72
Vision/hearing impairment	No impairment	1.3	1.0–1.6	0.05	1.3	0.9–1.8	0.20
History of stroke	No stroke	1.5	1.0–2.3	0.05	1.2	0.6–2.2	0.60
insomnia	Regular sleep	1.6	1.3–2.0	**< 0.001**	1.2	0.9–1.8	0.25
Renal dysfunction	Normal renal functions	1.7	1.1–2.5	**0.02**	1.7	1.0–2.7	**0.04**
Diabetes mellitus	Diabetes-free	1.2	0.9–1.6	0.29	–	–	–
BMI ≥ 25	BMI < 25	0.7	0.5–0.9	**< 0.01**	0.8	0.5–1.2	0.76
Postural hypotension	No postural hypotension	1.6	1.1–2.5	**0.02**	1.0	0.5–1.9	0.98

## Discussion

Owing to the aging global populations, cognitive impairment is increasingly becoming a pivotal societal challenge and a threat to sustainable development. Strong evidence supporting the cumulative deleterious effect of chronic arterial hypertension on cognitive function exists. Indeed, hypertension is the most important modifiable risk factor for cerebral white matter lesions, cognitive impairment, lacunar infarction, microbleeds, stroke, and vascular dementia[91, 92]. Pathophysiological mechanisms underpinning this complex yet intriguing association are not completely elucidated, however, summation of the cerebrovascular and degenerative lesions hypothesis is entertained[91, 92]. Nonetheless, with conflicting results between studies, the benefits of blood pressure control on cognitive functions among individuals with hypertension remains unclear[12, 43, 93–107]. Moreover, with the absence of effective disease-modifying treatments, hypertension being a modifiable risk factor represents a potentially vital mechanism for prevention or delay of cognitive impairment.

Over two-fifth of participants with hypertension in this present study had cognitive impairment. There is a wide variability in the prevalence of cognitive impairment (16.5–63.9%)[108–112] among persons with hypertension in the literature, however, our rate falls in between. Such discrepancy in the prevalence could be a result of the differences in population characteristics and variability in tools utilized for assessment of cognitive functions among studies. Nevertheless, comparative studies have consistently demonstrated superior rates of cognitive impairment to normotensive subjects[21, 110–112]. For instance, in a study by Muela HC et al.,[112] hypertensive individuals demonstrated twice as much prevalence of cognitive impairment compared to their normotensive counterparts (i.e. 50% vs 25%, *p* < 0.001). Furthermore, Muela[112] and colleagues revealed that patients with hypertension had worse performance in language, processing speed, visuospatial abilities, and memory upon neuropsychological tests. Likewise, in a study by Heizhat M et al.,[21] hypertensive individuals had significantly lower each item score and total score of the MMSE, compared to the normotensive controls.

A reciprocal interplay between education and cognitive functions has been observed across studies[113, 114]. Despite the lack of a formal consensus regarding the definition of low education, it is considered the most effective modifiable risk factor for cognitive impairment globally[115–117]. Recent analyses have noted that education does change the point at which accelerated declines due to cognitive impairment occur[118]. In this present study, participants with low education had over three-fold chance of having cognitive impairment and indeed it was the strongest predictor. These findings are consistent with a meta-analysis by Meng X and D’Arcy C which revealed a pooled OR of 2.61 for cognitive impairment among those with low education[119]. Educational attainment contributes to individual differences in cognitive skills hence people with higher education perform better across a broad range of cognitive tasks[120]. Moreover, current data shows that continuing education and cognitive leisure activities increase cognitive reserve thus improving cognitive functions and lower incidence of cognitive impairment[121].

The mechanism underlying the influence of rural-urban differences on cognitive functions is complex and poorly understood. Conversely, the impact of urbanization is profound and potentially mediated by several factors including education and occupation, living environments and pollution, access to public resources and healthcare, amongst others. Over the years and across all geographical boundaries, studies have demonstrated that individuals residing in rural areas have inferior cognitive functions compared to their age- and sex-matched urban counterparts[122–129]. In this study, persons who resided in rural areas had an 80% increased odds of having cognitive impairment compared to their urban counterparts. Such findings echo the one from a Taiwanese study by Liu CC et al. which revealed a 90% increased odds among rural dwellers.[130] Furthermore, studies by Chuang YF and Nakamura K revealed an odds of 2.3 and 4.0 respectively among rural residents[131, 132]. These regional differences in rates of cognitive impairment suggests the presence of modifiable factors with potential interventional implications, which ought to be elucidated in future studies.

A complex triad relationship between employment, cognition, and diseases exists. Through repetitive participation in demanding, complex tasks often requiring considerable focus and expertise, employment has the potential to augment cognitive reserve, facilitate brain health and optimize cognitive functioning as it entail learning of new skills, establishing a routine and social engagement[133–139]. The odds of cognitive impairment among unemployed/retired participants of this study was 1.7. In unison to our findings, a study by Leist AK et al.,[140] revealed an odds of 1.2 in unemployed individuals. Among unemployed/retired individuals, negative neuroplasticity with resultant compromise of cognitive functioning could ensue from the lack of cognitive stimulation provided from employment engagement. Moreover, owing to its potential in leading to effective cognitive interventions and in order to promote successful cognitive aging, it is imperative for clinicians to consider educating patients about the importance of staying cognitively active regardless of their employment status.

Epidemiologic data suggest that individuals at all stages of renal dysfunction have increased risk of developing cognitive disorders[141–153]. Consistent with previous research, this present study demonstrated a 70% increased likelihood of cognitive impairment among participants with renal dysfunction compared to their counterparts with preserved renal functions. Despite of its poorly understood pathophysiology, the relationship between cognitive impairment and renal dysfunction appears to be complex and bidirectional[154–156]. Nevertheless, direct neuronal injury from uremic toxins and the high prevalence of both subclinical and symptomatic ischemic cerebrovascular lesions are conceivable underlying mechanisms[157]. It is postulated that, for every decrease of 15 ml/min/1.73 m^2^in glomerular filtration rate, there is a decline in cognitive function similar to that of a 3-year aging[145]. Furthermore, cognitive impairment in individuals with renal dysfunction is associated with poor health-related quality of life, longer hospitalizations and higher mortality[158].

### Strengths and limitations

Several strengths can be drawn from this study including; (i) an adequate sample to estimate the prevalence of cognitive impairment and conduct analyses stratified according to potential effect modifiers, (ii) the use of standardized tools for data collection and utilization of qualified and competent personnel in all measurements, (iii) enrolled patients were attended in a tertiary hospital with a national status receiving cases from the whole country and thus our findings are perhaps generalizable to Tanzania and similar resource limited settings. Nevertheless, this study is not short of limitations. As this was the first local study to assess cognitive impairment, there was no normative data for comparison thus our cognitive impairment estimates could be over- or under-estimated. Lack of a non-hypertensive group for comparison prevents conclusions about whether cognitive impairment is linked to hypertension itself. Moreover, participants attending this tertiary level hospital may be systematically different to those attending lower level healthcare centers and thus our findings may not be generalizable to all populations in Tanzania. Furthermore, owing to the cross-sectional design, this study cannot preclude bias (i.e. referral filter bias) and limits both causality exploration and generalizability of findings. To elucidate the true nature and magnitude of this intriguing association, prospective studies are required to explore the longitudinal association between hypertension and incidence of cognitive impairment.

## Conclusion

In conclusion, this present study underscore that cognitive decline is highly prevalent among individuals with systemic hypertension. In view of this, it is pivotal to incorporate cognitive assessment in routine evaluation of hypertensive patients.

## Supplementary Information

**Additional file 1.**

**Additional file 2.**

## Data Availability

The datasets used and/or analyzed during the current study available from the corresponding author on reasonable request.

## Abbreviations

95% CI: 95% Confidence Interval
AMT: Abbreviated Mental Test
BMI: Body mass index
BP: Blood pressure
CKD: Chronic kidney disease
DBP: Diastolic blood pressure
eGFR: Estimated glomerular filtration rate
FBG: Fasting blood glucose
GPCOG: General Practitioner Assessment of Cognition Score
Hb: Hemoglobin
HIV: Human immunodeficiency virus
IQR: Interquartile range
JKCI: Jakaya Kikwete Cardiac Institute
JKCI-IRB: Jakaya Kikwete Cardiac Institute -Institutional Review Board
LDL: Low-density lipoprotein
MDRD: Modification of Diet in Renal Disease equation
MMSE: Mini–Mental State Examination
OR: Odd Ratio
PAVS: Physical Activity Vital Sign Questionnaire
RBG: Random blood glucose
SBP: Systolic blood pressure
SD: Standard deviation
WHO: World Health Organization
