# Supplementary material for: Burden and correlates of cognitive impairment among hypertensive patients in Tanzania: a cross-sectional study
Source: BMC Neurol. 2021 Nov 8;21:433. doi: 10.1186/s12883-021-02467-3 (PMC8573988; doi:10.1186/s12883-021-02467-3)
Supplement: Supplementary file 1 — Additional file 1. [file 12883_2021_2467_MOESM1_ESM.doc]

16^th^ June, 2021

Dear Editor,

Please find the enclosed manuscript entitled “**Burden and correlates of cognitive impairment among hypertensive patients in Tanzania: A cross-sectional study.**” to consider for publication in the BMC Neurology Journal.

Owing to the rapidly rising burden of hypertension in Tanzania, we sought to assess the prevalence and correlates of cognitive impairment among hypertensive patients attending a tertiary cardiovascular hospital in Tanzania. A total of 1201 hypertensive patients were enrolled in this study. The mean age was 58.1 years and females constituted nearly two-thirds of the study population. About three quarters had excess body weight, 16.6% had diabetes, 7.7% had history of stroke, 5.7% had heart failure, 16.7% had renal dysfunction, 53.7% had anemia, 27.7% had hypertriglyceridemia, 38.5% had elevated LDL, and 2.4% were HIV-infected. Nearly two-thirds of participants had uncontrolled blood pressure and 8.7% had orthostatic hypotension. Overall, 524 (43.6%) of participants had cognitive impairment. During bivariate analysis in a logistic regression model of 16 characteristics, 14 parameters showed association with cognitive functions. However, after controlling for confounders, multivariate analysis revealed ≤primary education (OR 3.5, 95%CI 2.4-5.2, p<0.001), unemployed state (OR 1.7, 95%CI 1.2-2.6, p<0.01), rural residence (OR 1.8, 95%CI 1.1-2.9, p=0.01) and renal dysfunction (OR 1.7, 95%CI 1.0-2.7, p= 0.04) to have independent association with cognitive decline.

We concluded that; that cognitive decline is highly prevalent among individuals with systemic hypertension. Moreover, our observation suggests that low education, rural life, unemployment status and renal dysfunction interactions with elevated blood pressure has the potential to predict cognitive performance over time. These findings highlight the necessity to improve hypertension diagnosis and optimize therapeutics so as to attain the target of cognitive decline prevention.

We hope you find this study worthy of publication in your esteemed journal.

Sincerely yours,

Pedro Pallangyo, M.D., M.P.H.
